# Effect of maternal job strain during pregnancy on infant neurodevelopment by gender at 6 and 12 months: Mothers and Children’s Environmental Health (MOCEH) study

**DOI:** 10.1186/s40557-015-0059-y

**Published:** 2015-03-20

**Authors:** Eunjeong Kim, HyeSook Park, Yun-Chul Hong, Mina Ha, Yangho Kim, Bo-Eun Lee, Eun-Hee Ha

**Affiliations:** Department of Preventive Medicine, Ewha Medical Research Center, Ewha Womans University, Seoul, South Korea; Department of Preventive Medicine, College of Medicine, Seoul National University College of Medicine, Seoul, South Korea; Department of Preventive Medicine, College of Medicine, Dankook University, Cheonan, South Korea; Department of Occupational and Environmental Medicine, Ulsan University Hospital, University of Ulsan College of Medicine, Ulsan, South Korea; Environmental Health Research Division, National Institute of Environmental Research, Incheon, South Korea

**Keywords:** Cognition, Job strain, Infant neurodevelopment, Prenatal psychosocial stress

## Abstract

**Objectives:**

Limited evidence is available regarding the association between prenatal job strain and infant neurodevelopment. Most studies used stress indicators other than job strain to explain the relationship between prenatal maternal stress and child development. The objective of this study was to investigate the association between maternal job strain during pregnancy and neurodevelopment in infancy.

**Methods:**

Mothers and Children’s Environmental Health (MOCEH) study, an on-going prospective birth cohort study, has been conducted in South Korea since 2006. Job strain during pregnancy was measured using Korean version of Job Content Questionnaire (JCQ). Infant neurodevelopment was assessed using Korean Bayley Scale of Infant Development II (K-BSID-II) at 6 and 12 months of age. A total of 343 mother-child pairs that completed JCQ and K-BSID-II more than once were included. Mental Developmental Index (MDI) and Psychomotor Developmental Index (PDI) defined in the K-BSID-II were used as outcome variables.

**Results:**

Compared to infants from mothers with low job strain, significant (*p* < 0.05) decreases in PDI were found in infants from mothers with active and passive job at 6 months of age. After stratification by infant sex, boys in the high strain group had a lower MDI score than boys in the low job strain group at 12 months. On the other hand, girls in the high strain and active groups had higher MDI scores than girls in the low job strain group at 12 months. PDI at 12 months also showed different results by sex. Boys in the high strain and passive job groups had lower PDI scores than boys in the low job strain group. However, such difference was not observed in girls.

**Conclusions:**

The findings of this study suggest that prenatal job strain affects infant neurodevelopment in a gender-dependent manner.

## Introduction

Traditionally, it is thought that a pregnant woman’s emotion and nutrition would influence fetal development. Therefore, good physical and psychosocial environment during pregnancy is required for optimal prenatal care [1]. Scientific evidence indicates that fetal environment is related to health during childhood and adulthood. Cardiovascular diseases and birth outcomes have been reported to be influenced by fetal programming [2-4].

One of the remarkable differences between pregnant Korean women in the present compared to them in the past might be that more women spend their pregnancy at work. The labor force participation rate of Korean women in their twenties and thirties has been increasing. In 2000, the rate of working Korean women was 54.9% for those in their twenties and 52.5% for those in their thirties. In 2013, these rates were increased to 62.0% and 57.0%, respectively. Since 2012, the labor force participation rate among women in their twenties has been higher than that in men [5]. Therefore, the number of women who work during pregnancy might have also increased. Although there is no official labor rate of women during pregnancy, interest in the effects of one’s job environment and job strain during pregnancy on the health of the offspring is growing [6]. Considering that average annual working hours in Korea (2163 in 2013) are much higher than the working hours in other OECD countries (1770, average hours in 2013) and that Korea ranks the second among OECD countries [7], job strain would be one of the main psychosocial stress factors in working women during pregnancy in Korea.

As the number of women who continue to work during pregnancy increase, some researchers have suggested that work stressor might adversely affect the offspring. Most studies have described the possible impact of mother job strain during pregnancy on their offspring in association with adverse birth outcomes, such as preterm delivery and low birth weight [8,9]. A Korean research study in 2011 reported that 39% of Korean women doctors answered that they had experienced adverse reproductive problems such as pregnancy complications, abortions, and infertility associated with stress at work [10]. In our previous studies, we also found a possible association between high job strain in pregnancy and low birth weight [6]. Epidemiologic research studies regarding prenatal maternal stress using general stress indicators (such as anxiety, depression, life events experienced, pregnancy-specific stress, and physiological indicators such as cortisol level) have detected a similar association between high levels of prenatal maternal stress and adverse birth outcomes, such as low birth weight [11-13] and preterm delivery [12,14-16].

Although occupational stress during pregnancy has been suggested as a potential contributor to adverse birth outcomes, limited studies have investigated the possible effect of prenatal work stressor on offspring’s further development and health. A birth cohort in Denmark suggested an association between maternal exposure to high strain with active jobs during pregnancy and asthma and atopic dermatitis in 7-year-old children [17].

Similar to birth outcomes, some researchers have studied the impact of maternal stress during pregnancy on children’s neurodevelopment, behavioral and emotional changes in children using general stress indicators or biologic response to stress [18-28]. However, evidence on the association between maternal working environment during pregnancy and infant neurodevelopment is sparse. Therefore, the objective of this study was to investigate the association between maternal job strain during pregnancy and infant neurodevelopment.

## Materials and methods

### Study design and participants

The present study represented part of the MOCEH (Mothers and Children’s Environmental Health) Study, a multiregional prospective birth cohort study conducted in South Korea. The study was established in 2006 to identify environmental factors that might affect the health statuses of mothers and children. The aim and structure of the MOCEH study have been described previously [29]. The regional centers involved in the study were located in Seoul, Cheonan, and Ulsan. Pregnant women residing in these cities over age of 18 were enrolled in their first trimester. All study participants provided written informed consent beforehand. This study was approved by the Institutional Review Boards of Ewha Womans University, Dankook University Hospital, and Ulsan University Hospital.

Between 2006 and 2010, 1,441 mothers of 1,751 mothers of singletons were enrolled in the MOCEH study. They responded to a questionnaire including question ‘Do you have a job now?’ in early or mid-pregnancy. Of these, 537 mothers answered that they had a job, including 496 who completed a questionnaire on job strain. A total of 373 infants of 496 mother-child pairs completed neurodevelopmental test once or twice when infants had reached the ages of 6 (6.5 ± 0.7) months and 12 (12.6 ± 0.9) months. Children with low birth weight (<2500 g) or with a preterm birth (gestational age of < 37 weeks) were excluded (n = 16). We also excluded mother-child pairs with missing data (n = 14). Ultimately, 343 pairs were included in the final analysis. Their average age was 30.1 years old. The percentage of male infants was 51.0%. Average birth weight was 3.33 kg. The average gestational age was 39.2 weeks. For reference, the average age of mothers in 2007 in South Korea was 30.6 years old. The average birth weight was 3.24 kg. The percentage of male infants was 51.5%. A total of 66.7% of babies were born at more than 39 gestational weeks while 5.2% of babies were born under 37 gestational weeks [30]. The average birth weight in this study was higher than the average level in Korea. This is probably due to the exclusion criteria of low birth weight used in this study.

### Neurodevelopment test

Infant neurodevelopment was assessed using the Korean version of the Bayley Scale of Infant Development II (K-BSID-II) [31], a standardized tool to assess toddler and infant development [32]. The Mental Developmental Index (MDI) and Psychomotor Developmental Index (PDI), composite scores of developmental performance of infants at different ages, were obtained from the K-BSID-II to compare performance distributions with the general population. They were used as independent variables. MDI and PDI were treated as continuous variables in a general linear model (GLM) and as dichotomous variables in a logistic regression model classified as at-risk/delayed (<85) or normal/above normal (≥85) [32]. As previously described [33], testing was conducted by trained examiners at each center in a quiet room. To ensure inter-rater reliability, rater training sessions and evaluation of the examinations by video monitoring were conducted annually.

### Measurement of job strain

Job strain was measured using the Korean version of the Job Content Questionnaire (JCQ). JCQ is a self-administered questionnaire for psychosocial job assessment based on the demand-control model devised by Karasek [34]. The demand-control model is a well-known method used to measure the social and psychological situation at work. Combining job demand and control results, this model estimates the different level of stress related to work. The psychological job demands (job demand) subscale consisted of 5 four-point scale items. The psychological job decision latitude (job control) consisted of 9 four-point scale items in the JCQ format in this study. The calculation of the job demand and control scales was based on the “JCQ User’s Guide” [35]. Physical job demands were measured using a single four-point scale items. Based on the answers of scale items, we calculated job demand and job control scale scores at their median values of our study and divided our subjects into the following four groups: low job strain group (low job demand and high job control), high job strain group (high job demand and low job control), active job group (high job demand and high job control), and passive job group (low job demand and low job control). We treated the low job strain group as a reference group and compared their children’s neurodevelopment indices with the other three groups that had higher job strain.

### Statistical analysis

Analysis of variance (ANOVA) was used to compare differences between the characteristics of study subjects. A linear regression model was used to explore the association between JCQ subscale scores (skill discretion, decision authority, decision latitude, psychological demands, physical demands) and MDI/PDI values. Differences between group neurodevelopment scores and developmental delays were analyzed using the general linear model (GLM) and logistic regression model, respectively.

Potential confounders related to child neurodevelopment were identified through literature search. The confounders were sociodemographic information such as maternal age, maternal education, and family income. They were collected from questionnaires completed at the first visit. Birth outcomes including birth weight, sex, and gestational age were obtained from medical records at birth. SAS (version 9.3; SAS Institute Inc. Cary, NC) was used for statistical analysis. All p-values were two-tailed. Statistical significance was considered when *p* value was less than 0.05.

## Results

The characteristics of participants by infant neurodevelopment indices at 6 and 12 months of age are summarized in Table 1. Infants of older women (>30 years) had lower neurodevelopment scores (MDI and PDI: *p* < 0.1) at 6 months. Boys had lower neurodevelopment scores than girls at 6 months (MDI: *p* < 0.1; PDI: *p* < 0.01). Characteristics of participants by maternal JCQ indices are provided in Table 2. Dichotomizing by median index levels, mothers in higher decision latitude group were older, had more education, and had a higher family income than mothers in the lower decision latitude group. The average birth weight was higher in the higher latitude group than that in the lower latitude group. In the lower physical job demand group, the average birth weight and gestational age were higher than in the higher physical job demand group.

**Table 1:** **Mean and standard deviation of neurodevelopment indices by infant age and participants’ characteristics (n = 343)** ^***a***^

**Characteristic**	**n (%) or mean ± SD** ^***b***^	**6 months (n = 306)**				**12 months (n = 252)**			
		**MDI**	**P-value**	**PDI**	**P-value**	**MDI**	**P-value**	**PDI**	**P-value**
**Total**		98.0 ± 10.9		97.0 ± 13.9		101.3 ± 15.9		94.6 ± 16.2	
**Maternal age**	30.1 ± 3.3								
≤30 years	208 (60.6)	98.9 ± 11.7	0.10	98.1 ± 14.1	0.08	100.5 ± 15.8	0.34	94.5 ± 15.5	0.89
>30 years	135 (39.4)	96.7 ± 9.3		95.2 ± 13.4		102.5 ± 16.1		94.8 ± 17.2	
**Maternal education**									
≤ High school	58 (16.9)	98.0 ± 11.0	1.00	98.5 ± 13.4	0.39	100.5 ± 14.0	0.75	95.0 ± 18.5	0.88
≥ University	285 (83.1)	98.0 ± 11.0		96.7 ± 13.9		101.4 ± 16.3		94.6 ± 15.7	
**Monthly family income**									
< $2,000	41 (12.0)	98.7 ± 9.2	0.78	98.7 ± 14.7	0.45	97.2 ± 17.4	0.10	96.0 ± 18.8	0.61
$2,000-$4,000	165 (48.1)	98.3 ± 11.9		97.5 ± 14.1		100.5 ± 15.2		93.7 ± 16.3	
> $4,000	137 (39.9)	97.5 ± 10.0		95.8 ± 13.2		103.7 ± 16.2		95.4 ± 15.1	
**Sex**									
Male	175 (51.0)	97.0 ± 10.9	0.09	94.6 ± 14.4	0.002	99.7 ± 16.5	0.11	93.4 ± 16.6	0.22
Female	168 (49.0)	99.1 ± 10.9		99.5 ± 12.9		102.9 ± 15.1		95.9 ± 15.6	
**Birth Weight (kg)**	3.33 ± 0.35								
**Gestational age (weeks)**	39.2 ± 1.0								

MDI: Mental Development Index, PDI: Psychomotor Development Index.

^*a*^Total number of mother-child pairs that underwent JCQ during pregnancy for a mother and K-BSID-II at 6 or 12 months of age for a child.

^*b*^Values were numbers and percentages for categorical variables and means ± SDs for continuous variables.

**Table 2:** **Distribution of JCQ indices by participants’ characteristics**

**Characteristic**	**Decision latitude (Job control)** ^***a***^		**Psychological job demands (Job demand)** ^***a***^		**Physical job demand** ^***b***^	
	**Total**		**Total**		**Total**	
	**Low (n(%))**	**High (n(%))**	**Low (n(%))**	**High (n(%))**	**Low (n(%))**	**High (n(%))**
**Number of participants**	158 (46.1)	185 (53.9)	154 (44.9)	189 (55.1)	217 (63.3)	126 (36.7)
**Maternal age** ^***c***^	29.4 ± 3.1	30.7 ± 3.4	30.0 ± 3.4	30.2 ± 3.3	30.2 ± 3.2	29.9 ± 3.6
P-value	<0.001		0.52		0.40	
≤30 years	106 (67.1)	102 (55.1)	90 (58.4)	118 (62.4)	126 (58.1)	82 (65.1)
>30 years	52 (32.9)	83 (44.9)	64 (41.6)	71 (37.6)	91 (41.9)	44 (34.9)
P-value	0.02		0.45		0.20	
**Maternal ** **Education**						
≤ High school	33 (20.9)	25 (13.5)	28 (18.2)	30 (15.9)	33 (15.2)	25 (19.8)
≥ University	125 (79.1)	160 (86.5)	126 (81.8)	159 (84.1)	184 (84.8)	101 (80.2)
P-value	0.07		0.57		0.27	
**Monthly family income**						
< $2,000	22 (13.9)	19 (10.3)	20 (13.0)	21 (11.1)	26 (12.0)	15 (11.9)
$2,000-$4,000	90 (60.0)	75 (40.5)	81 (52.6)	84 (44.4)	106 (48.9)	59 (46.8)
< $4,000	46 (29.1)	91 (49.2)	53 (34.4)	84 (44.4)	85 (39.2)	52 (41.3)
P-value	<0.001		0.17		0.92	
**Sex**						
Male	74 (46.8)	101 (54.6)	75 (48.7)	100 (52.9)	112 (51.6)	63 (50.0)
Female	84 (53.2)	84 (45.4)	79 (51.3)	89 (47.1)	105 (48.4)	63 (50.0)
P-value	0.15		0.44		0.77	
**Birth Weight (kg)** ^***c***^	3.28 ± 0.34	3.37 ± 0.35	3.33 ± 0.35	3.32 ± 0.35	3.36 ± 0.36	3.27 ± 0.32
P-value	0.02		0.82		0.03	
**Gestational age (weeks)** ^***c***^	39.1 ± 1.0	39.2 ± 1.1	39.3 ± 1.0	39.1 ± 1.1	39.2 ± 1.0	39.0 ± 1.1
P-value	0.67		0.23		0.07	

^*a*^Based on Job Control and Job Demand scores of JCQ – low: < 50th percentile, high: ≥ 50th percentile.

^*b*^Low: mothers who answered ‘strongly disagree’ or ‘disagree’ to the statement ‘my job requires lots of physical effort’.

High: mothers who answered ‘agree’ or ‘strongly agree’ in the same statement.

^*c*^Values were means ± SDs for continuous variables.

The relationships between the JCQ indices of mothers and neurodevelopmental indices at 6 and 12 months were determined using a linear regression model. No significant association was found (Table 3). Comparing the four job strain groups by MDI and PDI at 6 and 12 months using a general linear model (GLM), PDI at 6 months was significantly lower in the active job (β = −6.72, *p* = 0.001) and passive job (β = −7.86, *p* < 0.001) groups than in the reference low job strain group (mean: 102.0; 95% CI: 98.5 -105.5) (Tables 4 and 5). No association was found between maternal job strain and MDI at 6 or 12 months.

**Table 3:** **Association between maternal JCQ scores during pregnancy and infant neurodevelopment at 6 and 12 months** ^***a***^

	**6 months of age**												**12 months of age**											
	**MDI**						**PDI**						**MDI**						**PDI**					
**JCQ** ^***b***^	**Total**		**Boys**		**Girls**		**Total**		**Boys**		**Girls**		**Total**		**Boys**		**Girls**		**Total**		**Boys**		**Girls**	
	**Β**	**SE**	**β**	**SE**	**β**	**SE**	**β**	**SE**	**β**	**SE**	**β**	**SE**	**β**	**SE**	**β**	**SE**	**β**	**SE**	**β**	**SE**	**β**	**SE**	**β**	**SE**
**Skill Discretion**	0.09	0.13	0.11	0.17	0.002	0.19	0.07	0.16	0.11	0.23	−0.02	0.22	0.12	0.21	−0.05	0.30	0.31	0.30	0.17	0.21	0.16	0.30	0.21	0.32
P value	0.50		0.53		0.99		0.66		0.62		0.94		0.56		0.88		0.30		0.43		0.59		0.50	
**Decision Authority**	−0.04	0.10	−0.14	0.14	0.03	0.14	0.007	0.12	0.06	0.19	−0.05	0.16	0.03	0.15	−0.11	0.24	0.13	0.19	0.10	0.16	0.10	0.25	0.12	0.20
P value	0.66		0.32		0.83		0.95		0.74		0.75		0.83		0.65		0.49		0.52		0.69		0.56	
**Decision Latitude**	0.005	0.07	−0.03	0.10	0.02	0.10	0.02	0.09	0.06	0.13	−0.03	0.11	0.05	0.11	−0.06	0.16	0.15	0.14	0.10	0.11	0.09	0.17	0.12	0.15
P value	0.95		0.75		0.87		0.78		0.61		0.79		0.65		0.70		0.31		0.38		0.57		0.45	
**Psychological Demands**	0.07	0.13	−0.19	0.18	0.32	0.18	−0.02	0.16	−0.13	0.24	0.06	0.21	0.06	0.21	−0.38	0.31	0.39	0.27	0.02	0.21	−0.09	0.32	0.09	0.29
P value	0.56		0.29		0.08		0.89		0.59		0.77		0.77		0.23		0.16		0.93		0.78		0.76	
**Physical Demands**	0.001	0.75	−0.58	1.08	0.71	1.05	0.32	0.93	1.14	1.78	1.97	1.69	1.69	1.22	0.75	1.41	0.20	1.21	−0.12	1.27	0.26	1.81	−0.72	1.82
P value	1.00		0.59		0.50		0.73		0.52		0.25		0.17		0.60		0.87		0.92		0.89		0.69	

^*a*^Multiple regression model was adjusted for sex, birth weight, maternal age, gestational age, maternal education, and family income.

^*b*^The JCQ indices were considered continuous variables.

**Table 4:** **Association between maternal job strain during pregnancy and infant MDI stratified by sex** ^***a***^

**Time**	**Group**	**MDI**														
		**Total**					**Boys**					**Girls**				
		**N**	**Mean** ^***b***^	**β**	**SE**	**P value**	**N**	**Mean** ^***b***^	**β**	**SE**	**P value**	**N**	**Mean** ^***b***^	**β**	**SE**	**P value**
**6 months of age**	High strain	77	99.6	0.79	1.79	0.66	35	98.4	−0.31	2.68	0.91	42	101.1	2.14	2.47	0.39
	Active jobs	90	97.6	−1.25	1.71	0.47	54	95.9	−2.85	2.34	0.23	36	99.5	0.61	2.55	0.81
	Passive jobs	64	96.1	−2.76	1.91	0.15	30	96.5	−2.16	2.78	0.44	34	95.8	−3.10	2.67	0.25
	Low strain^*c*^	75	98.8	Ref.	-	-	38	98.7	Ref.	-	-	37	98.9	Ref.	-	-
**12 months of age**	High strain	66	99.9	−0.16	2.94	0.96	35	96.4	−9.46	4.43	0.03	31	102.5	8.25	3.88	0.04
	Active jobs	71	100.6	0.51	2.86	0.86	41	98.6	−7.30	4.08	0.08	30	103.0	8.74	3.94	0.03
	Passive jobs	60	100.5	0.47	3.02	0.88	27	101.9	−3.98	4.61	0.39	33	99.8	5.56	3.86	0.15
	Low strain^*c*^	55	100.1	Ref.	-	-	28	105.8	Ref.	-	-	27	94.2	Ref.	-	-

^*a*^The general linear model was used to find the association adjusted for sex, birth weight, maternal age, gestational age, maternal education, and family income.

^*b*^Adjusted means for sex, birth weight, maternal age, gestational age, maternal education, and family income.

^*c*^Reference group (Ref.).

**Table 5:** **Association between maternal job strain during pregnancy and infant PDI stratified by sex** ^***a***^

**Time**	**Group**	**PDI**														
		**Total**					**Boys**					**Girls**				
		**N**	**Mean** ^***b***^	**β**	**SE**	**P value**	**N**	**Mean** ^***b***^	**β**	**SE**	**P value**	**N**	**Mean** ^***b***^	**β**	**SE**	**P value**
**6 months of age**	High strain	77	99.9	−2.12	2.17	0.33	35	97.1	−4.94	3.40	0.15	42	102.3	0.20	2.79	0.94
	Active jobs	90	95.3	−6.72	2.08	0.001	54	94.5	−7.53	2.98	0.01	36	96.1	−6.01	2.88	0.04
	Passive jobs	64	94.2	−7.86	2.32	<0.001	30	92.5	−9.48	3.53	0.008	34	95. 9	−6.23	3.02	0.04
	Low strain^*c*^	75	102.0	Ref.	-	-	38	102.0	Ref.	-	-	37	102.1	Ref.	-	-
**12 months of age**	High strain	66	93.6	−4.16	3.00	0.17	35	91.4	−9.18	4.49	0.04	31	94.3	−0.45	4.24	0.92
	Active jobs	71	96.9	−0.80	2.93	0.79	41	96.2	−4.37	4.13	0.29	30	97.8	3.04	4.30	0.48
	Passive jobs	60	93.2	−4.51	3.09	0.15	27	91.5	−9.11	4.68	0.05	33	94.7	−0.02	4.22	1.00
	Low strain^*c*^	55	97.7	Ref.	-	-	28	100.6	Ref.	-	-	27	94.7	Ref.	-	-

^*a*^The general linear model was used to find the association adjusted for sex, birth weight, maternal age, gestational age, maternal education, and family income.

^*b*^Adjusted means for sex, birth weight, maternal age, gestational age, maternal education, and family income.

^*c*^Reference group (Ref.).

After putting the interaction term ‘infant sex and maternal job strain groups’ in the GLM analysis, a significant interaction was found between maternal job strain and MDI at 12 months (high strain group: *p* = 0.003; active job group: *p* = 0.004). To explore gender effects, we additionally described the results of stratified analysis by infants’ sex (Tables 4 and 5). Although no association was found between job strain and MDI at 6 or 12 months without stratification, boys in the high strain group (β = −9.46, *p* = 0.03) had lower MDI scores than boys in the reference group at 12 months. On the other hand, girls in high strain (β = 8.25, *p* = 0.04) and active job (β = 8.74, *p* = 0.03) groups had higher MDI scores than girls in the reference group at 12 months.

At 6 months in boys and girls, PDI values were similar to the results before the stratification. PDI at 6 months was lower in the active job group (boys β = −7.53, *p* = 0.01; girls β = −6.01, *p* = 0.04) and in the passive job group (boys β = −9.48, *p* = 0.008; girls β = −6.23, *p* = 0.04) than that in the reference group. However, PDI at 12 months showed different results by sex. In boys, the high strain (β = −9.18, *p* = 0.04) and the passive job (β = −9.11, *p* = 0.05) groups had lower PDI scores than the reference group. However, such difference was not observed in girls.

MDI and PDI were also converted to dichotomous variables to understand the association between maternal job strain and infant developmental delay using a logistic regression model. No significant association was found in the model (Table 6). Because no significant interaction was found by infants’ sex in this model and the small number of prevalence in infant developmental delay by gender might lower the statistical power, further stratification by gender was not performed in the logistic regression analysis.

**Table 6:** **Association between maternal job strain during pregnancy and neurodevelopment delay in infants** ^***a***^

		**MDI** ^***b***^				**PDI** ^***b***^			
**Time**	**Group**	**N** ^***c***^ **(%)**	**OR**	**LCI** ^***d***^	**UCI** ^***d***^	**N** ^***c***^ **(%)**	**OR**	**LCI** ^***d***^	**UCI** ^***d***^
**6 months of age**	High strain	7/77(9.1%)	1.288	0.403	4.115	8/77(10.4%)	1.049	0.362	3.037
	Active jobs	11/90(12.2%)	1.506	0.521	4.352	20/90(22.2%)	2.383	0.968	5.867
	Passive jobs	11/64(17.2%)	2.562	0.852	7.707	13/64(20.3%)	2.643	0.973	7.178
	Low strain^*e*^	6/75(8.0%)	Ref.	-	-	8/75(10.7%)	Ref.	-	-
**12 months of age**	High strain	10/66(15.2%)	1.125	0.378	3.347	22/66(33.3%)	2.176	0.904	5.237
	Active jobs	6/71(8.5%)	0.569	0.175	1.844	20/71(28.2%)	1.705	0.714	4.068
	Passive jobs	8/60(13.3%)	1.077	0.345	3.365	20/60(33.3%)	2.406	0.980	5.908
	Low strain^*e*^	7/55(12.7%)	Ref.	-	-	10/55(18.2%)	Ref.	-	-

^a^Logistic regression model was used to find the association adjusted for sex, birth weight, maternal age, gestational age, maternal education, and family income.

^*b*^MDI and PDI were treated as dichotomous variables, classified as at-risk/delayed (<85) or normal/above normal (≥85).

^*c*^The number of children with MDI or PDI score under 85/total number in each group.

^*d*^95% lower confidence interval (LCI) and upper confidence interval (UCI).

^*e*^Reference group (Ref.).

## Discussion

In the current study, we examined the relationship between maternal job strain during pregnancy and infant neurodevelopment at 6 and 12 months. In boys at 12 months, high job strain during pregnancy was found to have lower cognitive development (MDI) and psychomotor development (PDI). However, the association of job strain during pregnancy with MDI was opposite in girls at 12 months. Compared to girls from mothers in the low strain group, girls from mothers in the high strain group showed higher MDI scores at 12 months. Furthermore, we found lower PDI in passive and active job group among both boys and girls at 6 months compared to children in the low strain group. The differences of MDI and PDI among the four groups were within the normal range of infant development (≥85). No significant differences of developmental delay were found among the four groups.

Previous studies regarding the effect of work related stress during pregnancy primarily examined adverse birth outcomes, such as preterm delivery and low birth weight [6,8,36]. To the best of our knowledge, the relationship between maternal job stress during pregnancy and infant neurodevelopment has not been previously studied. Outside of work stressor, some researchers have described an association between prenatal psychological stress and neurodevelopment in early childhood. A Belgian cohort study has suggested that maternal anxiety is correlated with fetal behavior, which might influence neonatal and infant behavior [37]. Another study conducted in the Netherlands revealed a significant negative association between maternal anxiety and MDI at age 2 but no association at age 1 [38]. In another study, children of women who experienced major storm during pregnancy had lower MDI scores at age 2 [25]. After examining the effect of maternal stress and cortisol levels during pregnancy on infant neurodevelopment at 3 and 8 months, negative associations between maternal stress and infant MDI/PDI at 8 months and between maternal cortisol levels and infant MDI/PDI at 3 months were found [24].

The hypothalamic-pituitary-adrenal (HPA) axis model is one of the most explained mechanisms with respect to the association between prenatal maternal stress and child outcomes [39]. The level of cortisol, the end product of this axis, changes with maternal stress. In addition, cortisol can cross the placental barrier and affect fetal development. Maternal stress might also alter the regulation of 11β-HSD2 in the placenta, which controls fetal exposure to cortisol by converting active cortisol into inactive cortisone [40,41]. Although the HPA axis model is usually used to explain the association, available evidence is not convincing. Other alternative mechanisms involving the autonomic nervous system (ANS) and uterine blood flow have been suggested. Stress can induce fetal sympathetic nervous system activity and alter ANS balance [42]. Increased adrenal hormone levels induced by stress can cause vasoconstriction in the uterus and hypoxic fetal condition [43].

However, maternal distress during pregnancy has not always been related to negative impact on children’s neurodevelopment. In one study that assessed maternal distress during mid-pregnancy, it was found that higher levels of prenatal anxiety, nonspecific stress, and depressive symptoms strengthened motor development at age 2 [1]. Anxiety and depression were also found to have positive effects on infant mental development in that study. Several authors reported that mild prenatal stress related to intrauterine conditions such as hypoxia and maternal hypertension improved the growth and development of offsprings [44-46]. Such fetal physiological adaptations could explain the contrary associations found in studies. Whether the levels of stress will have positive effects on offspring development remains controversial [1]. One study suggested that the impact of stressors during pregnancy on infant development might depend on the timing of exposure [23]. They found that a high MDI at age 1 was associated with not only a low level of maternal cortisol during early pregnancy but also with a high level of maternal cortisol at the end of pregnancy. These results suggest that maternal distress might bring different outcomes to children’s neurodevelopment depending on the timing of exposure during gestation, duration of the stress, features and strength of the stress, and other unknown factors.

The main difference between those studies and the present study is the measured stress indicator. High level of anxiety and depression or experience of stressful life events may reflect severe stress. Different aspect of stress from high strain at work defined by a combination of high job demands with low control over work might affect the result [17]. Therefore, job strain should be considered as a specific part of stressors. More studies related to maternal job strain during pregnancy will be needed to understand whether the findings are consistent with the present study. Additionally, because those epidemiological studies did not show relevant results regarding gender difference on infant neurodevelopment, it is difficult to directly compare their results with the present study.

In the present study, different effect of gender on infant cognitive and psychomotor development at 12 months of age was found. High job strain during pregnancy had a negative impact on MDI and PDI among boys at 12 months. However, in girls, high job strain was associated with an increase of MDI without significant difference of PDI at 12 months. Regarding sex-specific response to prenatal stress, there have been accumulating evidences regarding sex-specific effect. It was found that males were generally more sensitive to stress and maternal corticosteroids in uterus [47-50]. Although the biological mechanism remains unclear, sex-differences in glucocorticoid and mineralocorticoid receptors during brain development accounting for the fetal vulnerability have been found in males after fetal exposure to glucocorticoid [49].

The influence of gender on the relation between maternal stress and cognitive development in early childhood has rarely been mentioned in human studies. However, an animal study demonstrated the susceptibility of cognitive development to stress during pregnancy and found sex-specific effects on learning and avoiding strategies [51]. In other studies, while prenatal stress lowered the ability to learn in male offspring, the same stress did not affect female offspring. It even increased their learning performance [52-54], which is in consistent with our result. Gonadal hormones have been suggested to contribute to the observed differences. It has been suggested that prenatal stress might masculinize the female brain and help improve performance using a male-like cued strategy [51,55]. More epidemiologic studies are needed to determine whether such gender-dependent responses relevant to our results are consistently found in children.

Among four quadrants in the job-strain model, high strain was represented as the most stressful aspects at work condition. It would have the greatest impact on infants’ neurodevelopment. Unexpectedly, lower PDI in passive and active job group was found in both boys and girls at 6 months compared to children in the low strain group. However, they were not significantly different from the high strain group. Because the same results were not continuously shown in the results at 12 months, one considerable reason might be the effect of postnatal confounders at that period such as breast-feeding or a different type of fosterers. Small sample size might be another reason.

The strength of this study was that infant neurodevelopment tests were performed prospectively by trained raters. Thus, the prospective design might have protected against recall bias about maternal job strain during pregnancy. In addition, the study identified a sex-specific effect of maternal stress on child development, which has rarely been demonstrated in epidemiologic studies. Furthermore, this is the first study to evaluate the association between prenatal maternal job strain and infant neurodevelopment. Previous studies have mostly addressed the effect of job stress during pregnancy on birth outcomes [3,6].

On the other hand, the study has several limitations. First, the maternal job strain was assessed only once after enrollment, mostly in early or mid-pregnancy (before 20 weeks of pregnancy). It was not repeatedly evaluated during pregnancy or post-natal, although the exposure at different gestational weeks during pregnancy might affect fetal development in a different manner. Therefore, we could not adjust for the effect of the job environment during late pregnancy or the postnatal period to understand the sensitive time window of maternal job strain during the pregnancy period on infant neurodevelopment. Furthermore, considerable confounders - parity and breastfeeding - which might affect children’s neurodevelopment were not analyzed because of many missing values. Another study limitation is that the correlation between the maternal cortisol levels and job strain was not investigated, although this correlation has not always been found by others [3,23,56]. Moreover, job strain is a factor that reflects job distress in each working condition. However, it does not represent the mother’s non-occupational or general psychosocial distress. Further studies on possible effects of other stress factors such as life events on outcomes or on correlations between the stressors will help us understand better the association between job strain during pregnancy and children’s neurodevelopment. In addition, the subjects in this study were limited to women who had been working during their pregnancy and their characteristics might be different from pregnant women in the general population. Therefore, the association of prenatal job strain should not be understood as a general manner of prenatal stress. In addition, subjects’ job characteristics were not considered in the model, although they might influence the job stress level and the results. In this study, we used JCQ to understand job-related distress in different work settings in a general manner [57]. The sample size was relatively small compared to previous studies [58]. This might have lowered the statistical power.

## Conclusion

This study indicates that occupational stress at work during pregnancy probably might affect infant neurodevelopment. It shows that prenatal job strain has gender-associated effects on infant neurodevelopment and that boys might be more susceptible to prenatal psychosocial stress than girls. Further follow-up evaluation is needed in order to investigate the effect of prenatal job strain on older children.

## References

[1] DiPietro JA, Novak MF, Costigan KA, Atella LD, Reusing SP (2006). Maternal psychological distress during pregnancy in relation to child development at age two. Child Dev.

[2] Barker DJ, Eriksson JG, Forsén T, Osmond C (2002). Fetal origins of adult disease: strength of effects and biological basis. Int J Epidemiol.

[3] van Dijk AE, van Eijsden M, Stronks K, Gemke RJ, Vrijkotte TG (2012). The association between prenatal psychosocial stress and blood pressure in the child at age 5–7 years. PLoS One.

[4] Brunton PJ (2013). Effects of maternal exposure to social stress during pregnancy: consequences for mother and offspring. Reproduction.

[5] Economically Active Population Survey. [http://kosis.kr/statisticsList/statisticsList_01List.jsp?vwcd=MT_ZTITLE&parmTabId=M_01_01]

[6] Lee BE, Ha M, Park H, Hong YC, Kim Y, Kim YJ (2011). Psychosocial work stress during pregnancy and birthweight. Paediatr Perinat Epidemiol.

[7] Average annual hours actually worked per worker. [http://stats.oecd.org/Index.aspx]

[8] Mutambudzi M, Meyer JD, Warren N, Reisine S (2011). Effects of psychosocial characteristics of work on pregnancy outcomes: a critical review. Women Health.

[9] Niedhammer I, O’Mahony D, Daly S, Morrison JJ, Kelleher CC (2009). Group LC-GCSS: Occupational predictors of pregnancy outcomes in Irish working women in the Lifeways cohort. BJOG.

[10] Park K, Rhee Y, Moon BB, Park G, Won J: A Study on Marriage and Child Care Environment for Korean Women in Medicine. Republic of Korea; 2011

[11] Diego MA, Jones NA, Field T, Hernandez-Reif M, Schanberg S, Kuhn C (2006). Maternal psychological distress, prenatal cortisol, and fetal weight. Psychosom Med.

[12] Lobel M, Dunkel-Schetter C, Scrimshaw SC (1992). Prenatal maternal stress and prematurity: a prospective study of socioeconomically disadvantaged women. Health Psychol.

[13] Littleton HL, Bye K, Buck K, Amacker A (2010). Psychosocial stress during pregnancy and perinatal outcomes: a meta-analytic review. J Psychosom Obstet Gynaecol.

[14] Glynn LM, Schetter CD, Hobel CJ, Sandman CA (2008). Pattern of perceived stress and anxiety in pregnancy predicts preterm birth. Health Psychol.

[15] Dole N, Savitz DA, Siega-Riz AM, Hertz-Picciotto I, McMahon MJ, Buekens P (2004). Psychosocial factors and preterm birth among African American and White women in central North Carolina. Am J Public Health.

[16] Lobel M, Cannella DL, Graham JE, DeVincent C, Schneider J, Meyer BA (2008). Pregnancy-specific stress, prenatal health behaviors, and birth outcomes. Health Psychol.

[17] Larsen AD, Schlünssen V, Christensen BH, Bonde JP, Obel C, Thulstrup AM (2014). Exposure to psychosocial job strain during pregnancy and odds of asthma and atopic dermatitis among 7-year old children - a prospective cohort study. Scand J Work Environ Health.

[18] O’Connor TG, Heron J, Glover V, Team AS (2002). Antenatal anxiety predicts child behavioral/emotional problems independently of postnatal depression. J Am Acad Child Adolesc Psychiatry.

[19] O’Connor TG, Heron J, Golding J, Beveridge M, Glover V (2002). Maternal antenatal anxiety and children’s behavioural/emotional problems at 4 years. Report from the Avon Longitudinal Study of Parents and Children. Br J Psychiatry.

[20] Rice F, Jones I, Thapar A (2007). The impact of gestational stress and prenatal growth on emotional problems in offspring: a review. Acta Psychiatr Scand.

[21] Talge NM, Neal C, Glover V (2007). Early Stress TaRaPSNFaNEoCaAMH: Antenatal maternal stress and long-term effects on child neurodevelopment: how and why?. J Child Psychol Psychiatry.

[22] O’Connor TG, Monk C, Fitelson EM (2014). Practitioner Review: Maternal mood in pregnancy and child development - implications for child psychology and psychiatry. J Child Psychol Psychiatry.

[23] Davis EP, Sandman CA (2010). The timing of prenatal exposure to maternal cortisol and psychosocial stress is associated with human infant cognitive development. Child Dev.

[24] Huizink AC, de Medina PG R, Mulder EJ, Visser GH, Buitelaar JK (2003). Stress during pregnancy is associated with developmental outcome in infancy. J Child Psychol Psychiatry.

[25] Laplante DP, Barr RG, Brunet A, Galbaud duFort G, Meaney ML, Saucier JF (2004). Stress during pregnancy affects general intellectual and language functioning in human toddlers. Pediatr Res.

[26] Davis EP, Glynn LM, Schetter CD, Hobel C, Chicz-Demet A, Sandman CA (2007). Prenatal exposure to maternal depression and cortisol influences infant temperament. J Am Acad Child Adolesc Psychiatry.

[27] Huizink AC, de Medina PG, Mulder EJ, Visser GH, Buitelaar JK (2002). Psychological measures of prenatal stress as predictors of infant temperament. J Am Acad Child Adolesc Psychiatry.

[28] de Weerth C, van Hees Y, Buitelaar JK (2003). Prenatal maternal cortisol levels and infant behavior during the first 5 months. Early Hum Dev.

[29] Kim BM, Ha M, Park HS, Lee BE, Kim YJ, Hong YC (2009). The Mothers and Children’s Environmental Health (MOCEH) study. Eur J Epidemiol.

[30] Korea S. Annual Report on Live Births and Deaths Statistics 2007. 2008.

[31] Park K, Cho B (2006). Korean Bayley Scales of Infant Development. 2nd ed. Interpretation Manual.

[32] Bayley N (1993). Bayley Scales of Infant Development.

[33] Kim Y, Ha EH, Kim EJ, Park H, Ha M, Kim JH (2011). Prenatal exposure to phthalates and infant development at 6 months: prospective Mothers and Children’s Environmental Health (MOCEH) study. Environ Health Perspect.

[34] Eum KD, Li J, Lee HE, Kim SS, Paek D, Siegrist J (2007). Psychometric properties of the Korean version of the effort-reward imbalance questionnaire: a study in a petrochemical company. Int Arch Occup Environ Health.

[35] Job content questionnaire and user’s guide. [http://www.jcqcenter.org/JCQGuide_12885-Rev%201.pdf]

[36] Larsen AD, Hannerz H, Juhl M, Obel C, Thulstrup AM, Bonde JP (2013). Psychosocial job strain and risk of adverse birth outcomes: a study within the Danish national birth cohort. Occup Environ Med.

[37] Van den Bergh BRH (1990). The influence of maternal emotions during pregnancy on fetal and neonatal behavior. Pre-Peri-natal Psychol.

[38] Evelien PMB, van Baar AL, Pop VJM (2001). Maternal anxiety during pregnancy and subsequent infant development. Infant Behav Dev.

[39] Henry C, Kabbaj M, Simon H, Le Moal M, Maccari S (1994). Prenatal stress increases the hypothalamo-pituitary-adrenal axis response in young and adult rats. J Neuroendocrinol.

[40] Glover V, Bergman K, Sarkar P, O’Connor TG (2009). Association between maternal and amniotic fluid cortisol is moderated by maternal anxiety. Psychoneuroendocrinology.

[41] Barker DJ, Osmond C, Kajantie E, Eriksson JG (2009). Growth and chronic disease: findings in the Helsinki Birth Cohort. Ann Hum Biol.

[42] Field T, Diego M, Hernandez-Reif M (2006). Prenatal depression effects on the fetus and newborn: a review. Infant Behav Dev.

[43] Teixeira JM, Fisk NM, Glover V (1999). Association between maternal anxiety in pregnancy and increased uterine artery resistance index: cohort based study. BMJ.

[44] Huether G (1998). Stress and the adaptive self-organization of neuronal connectivity during early childhood. Int J Dev Neurosci.

[45] Allen MC, Donohue PK (2002). Neuromaturation of multiples. Semin Neonatol.

[46] Ponirakis A, Susman EJ, Stifter CA (1998). Negative emotionality and cortisol during adolescent pregnancy and its effects on infant health and autonomic nervous system reactivity. Dev Psychobiol.

[47] Bruckner TA, Catalano R, Ahern J (2010). Male fetal loss in the U.S. following the terrorist attacks of September 11, 2001. BMC Public Health.

[48] Clifton VL, Murphy VE (2004). Maternal asthma as a model for examining fetal sex-specific effects on maternal physiology and placental mechanisms that regulate human fetal growth. Placenta.

[49] Owen D, Matthews SG (2003). Glucocorticoids and sex-dependent development of brain glucocorticoid and mineralocorticoid receptors. Endocrinology.

[50] Bekedam DJ, Engelsbel S, Mol BW, Buitendijk SE, van der Pal-de Bruin KM (2002). Male predominance in fetal distress during labor. Am J Obstet Gynecol.

[51] Bowman RE, MacLusky NJ, Sarmiento Y, Frankfurt M, Gordon M, Luine VN (2004). Sexually dimorphic effects of prenatal stress on cognition, hormonal responses, and central neurotransmitters. Endocrinology.

[52] Bowman RE (2005). Stress-induced changes in spatial memory are sexually differentiated and vary across the lifespan. J Neuroendocrinol.

[53] Nishio H, Kasuga S, Ushijima M, Harada Y (2001). Prenatal stress and postnatal development of neonatal rats–sex-dependent effects on emotional behavior and learning ability of neonatal rats. Int J Dev Neurosci.

[54] Mueller BR, Bale TL (2007). Early prenatal stress impact on coping strategies and learning performance is sex dependent. Physiol Behav.

[55] Van Goozen SH, Cohen-Kettenis PT, Gooren LJ, Frijda NH, Van de Poll NE (1994). Activating effects of androgens on cognitive performance: causal evidence in a group of female-to-male transsexuals. Neuropsychologia.

[56] Himes KP, Simhan HN (2011). Plasma corticotropin-releasing hormone and cortisol concentrations and perceived stress among pregnant women with preterm and term birth. Am J Perinatol.

[57] Karasek R, Brisson C, Kawakami N, Houtman I, Bongers P, Amick B (1998). The Job Content Questionnaire (JCQ): an instrument for internationally comparative assessments of psychosocial job characteristics. J Occup Health Psychol.

[58] Tegethoff M, Greene N, Olsen J, Schaffner E, Meinlschmidt G (2011). Stress during pregnancy and offspring pediatric disease: A National Cohort Study. Environ Health Perspect.

